# Neutralizing Monoclonal Antibodies as Promising Therapeutics against Middle East Respiratory Syndrome Coronavirus Infection

**DOI:** 10.3390/v10120680

**Published:** 2018-11-30

**Authors:** Hui-Ju Han, Jian-Wei Liu, Hao Yu, Xue-Jie Yu

**Affiliations:** 1 School of Health Sciences, and State Key Laboratory of Virology, Wuhan University, Wuhan 430071, China; nikihuijuhan@163.com (H.-J.H.); liujw_2012@163.com (J.-W.L.); 2 Fudan University School of Medicine, Shanghai 200032, China; howardyu89@163.com

**Keywords:** Middle East Respiratory Syndrome Virus, MERS-CoV, neutralizing monoclonal antibodies

## Abstract

Since emerging in 2012, Middle East Respiratory Syndrome Coronavirus (MERS-CoV) has been a global public health threat with a high fatality rate and worldwide distribution. There are no approved vaccines or therapies for MERS until now. Passive immunotherapy with neutralizing monoclonal antibodies (mAbs) is an effective prophylactic and therapeutic reagent against emerging viruses. In this article, we review current advances in neutralizing mAbs against MERS-CoV. The receptor-binding domain (RBD) in the spike protein of MERS-CoV is a major target, and mouse, camel, or human-derived neutralizing mAbs targeting RBD have been developed. A major problem with neutralizing mAb therapy is mutant escape under selective pressure, which can be solved by combination of neutralizing mAbs targeting different epitopes. Neutralizing mAbs are currently under preclinical evaluation, and they are promising candidate therapeutic agents against MERS-CoV infection.

## 1. Introduction

Middle East Respiratory Syndrome (MERS) emerged in 2012 in Saudi Arabia with the death of a man with pneumonia; the causative agent was subsequently identified as MERS-CoV, which belonged to lineage C betacoronaviruses [1]. With dromedary camels (*Camelus dromedarius*, also known as Arabian camel) as direct sources and bats as potential reservoirs [2], MERS-CoV has been frequently introduced into human populations. Once MERS-CoV is introduced into a person, person-to-person transmission might occur, and is responsible for approximately 40% of MERS cases globally [3]. MERS-CoV has been a consistent threat to humans. As of October 2018, MERS-CoV has caused 2254 laboratory-confirmed human cases, including 800 deaths in 27 countries, with the fatality rate as high as 35% (http://www.who.int/emergencies/mers-cov/en/). Although MERS cases are primarily reported in the Middle East, facilitated by international travelling, MERS-CoV can also be a worldwide threat, which is well illustrated by the MERS outbreak in South Korea in 2015 [4]. Given the potential risk of causing worldwide public health emergencies and the absence of licensed vaccines and antiviral therapeutics, the World Health Organization has listed MERS-CoV in the “List of Blueprint priority diseases” (http://www.who.int/blueprint/priority-diseases/en/).

Vaccines are the most important approach against viral infections, but usually take a long time to develop. They are also unable to provide either immediate prophylactic protection or treat ongoing viral infections. Neutralizing monoclonal antibodies (mAbs) have recently emerged as a powerful tool to provide prophylactic and therapeutic protection against emerging viruses [5]. Potent neutralizing mAbs can be achieved by various technologies, such as hybridoma technology, humanized mouse, phage or yeast display, and single B cell isolation [5].

## 2. Spike (S) Protein of MERS-CoV as Target for Neutralizing mAbs

**Figure 1:**
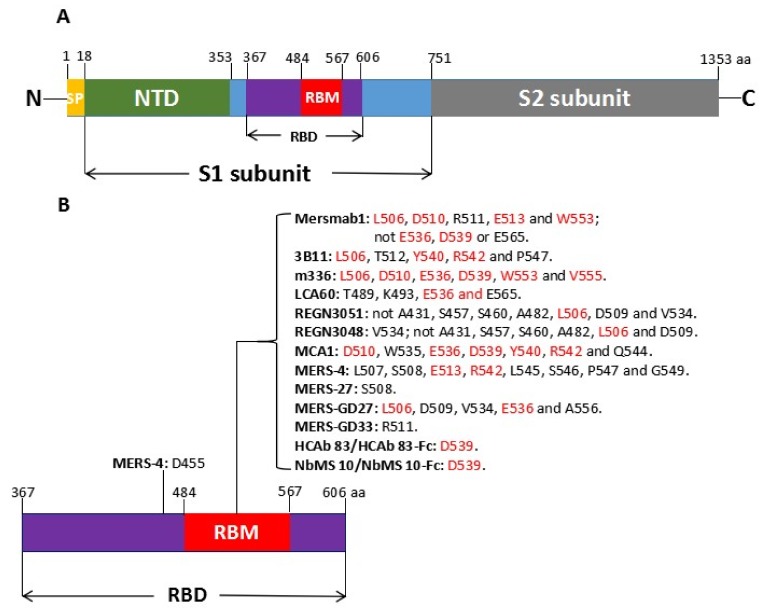
(**A**) Schematic representation of MERS-CoV S protein. (**B**) Residues on RBD critical for mAb neutralization. SP: signal peptide; NTD: N-terminal domain; RBD: receptor-binding domain; RBM: receptor-binding motif. Conserved residues on RBM critical for hDPP4 binding are shown in red.

MERS-CoV is a single, positive-stranded RNA virus of about 30 kb, which encodes four major viral structural proteins—including spike (S), envelope (E), membrane (M) and nucleocapsid (N)—as well as several accessory proteins [6]. The S protein (1353 aa) plays an important role in virus infection and consists of a receptor-binding subunit S1 (aa 18–751) and a membrane-fusion subunit S2 (aa 752–1353). S1 mediates viral attachment to host cells and S2 mediates virus-cell membrane fusion [7]. The S1 subunit contains a receptor-binding domain (RBD) (aa 367–606) [8] that can bind to cell receptor dipeptidyl peptidase 4 (DPP4, also known as CD26), and mediates viral attachment target cells [9]. The RBD consists of a core subdomain and a receptor-binding motif (RBM) (aa 484–567). The schematic representation of MERS-CoV S protein is shown in Figure 1A.

Neutralizing mAbs binding to the S protein of MERS-CoV can prevent viral attachment to the cell receptor and inhibit viral entry [7]. The S protein of MERS-CoV is a key target for antivirals, and RBD is the most popular focus. In this study, we review the current knowledge on neutralizing mAbs targeting the RBD of MERS-CoV.

## 3. Mouse Neutralizing mAbs

### 3.1. 4C2 and 2E6

Stable hybridoma cell lines were generated by fusing myeloma cells with splenocytes of mice that were immunized with MERS-RBD protein. Two neutralizing mAbs, 4C2 and 2E6, had high affinity for the RBD of MERS-CoV and blocked both pseudovirus and live MERS-CoV entry into cells with high efficacy [10]. Humanized 4C2 showed similar neutralizing activity in cell entry tests. In vivo tests indicated that 4C2 could significantly reduce the virus titers in the lungs of Ad5-hCD26-transduced mice which were infected with MERS-CoV, highlighting its potential application in humans not only for preventing but also treating MERS-CoV infection. Crystallization of the 4C2 Fab/MERS-RBD complex showed that the 4C2 recognized conformational epitopes (Y397-N398, K400, L495-K496, P525, V527-S532, W535-E536, and D539-Q544), which were partially overlapped the receptor-binding footprint in the RBD of MERS-CoV. The 4C2 complex interfered with MERS-CoV binding to DPP4 by both steric hindrance and interface-residue competition. 2E6 competed with 4C2 to bind to MERS-RBD, indicating that they recognized proximate or overlapping epitopes [10].

### 3.2. Mersmab1

Neutralizing mAb Mersmab1 was obtained by fusing myeloma cells with splenocytes of a mouse that was immunized with recombinant MERS-CoV S1 [11]. Mersmab1 effectively blocked the entry of pseudovirus and live MERS-CoV into cells. Structural analysis showed that Mersmab1 bound to the RBD of MERS-CoV through recognizing conformational epitopes, and all of the residues critical for Mersmab1 binding were located on the left ridge of RBM. Mersmab1 neutralized MERS-CoV by competitively blocking the binding of MERS-CoV RBD to DPP4. Based on escape mutant analysis of the key residues on the RBD, it was found that residue L506, D510, R511, E513, and W553 were critical for Mersmab1 binding to the RBD, while mutation of E536, D539, or E565 did not affect the interaction of Mersmab1 and the RBD at all [11].

## 4. Human Neutralizing mAbs

### 4.1. 3B11

An ultra-large nonimmune human antibody-phage display library was constructed with B cells of unimmunized donors. With a unique spanning strategy, seven human neutralizing mAbs with varying neutralization efficacy to MERS-CoV were identified [12]. Binding detection demonstrated that the epitopes of these mAbs lay within aa 349–590 of the S protein, which overlapped a large part of the RBD of MERS-CoV. Binding competition assays showed that these mAbs recognized at least three distinct epitope groups, which was further confirmed by escape studies. With no cross-epitope resistance, these mAbs neutralized MERS-CoV by competitively blocking the binding of the RBD of MERS-CoV to DPP4. Escape mutant assays showed that five residues were critical for neutralization of these mAbs, namely L506, T512, Y540, R542, and P547. Of the seven mAbs, 3B11 exhibited the best neutralization activity against both pseudovirus and live MERS-CoV infectivity in cells. Moreover, under the selective pressure of these mAbs, the IgG form of 3B11 was superior, since it did not induce neutralization escape [12]. In vivo tests demonstrated that 3B11 reduced lung pathology in rhesus monkeys infected with MERS-CoV [13]. With its high neutralizing activity and suppression of mutant escape, 3B11 in the IgG form is a promising therapeutic mAb against MERS-CoV.

### 4.2. m336

Three human mAbs—m336, m337, and m338—were identified from a large naïve human phage display antibody library, which was constructed with peripheral blood mononuclear cells from healthy volunteers [14]. The binding sties of the three mAbs were within the RBD of MERS-CoV (aa 377–588), therefore they neutralized MERS-CoV by competing with DPP4 binding to the RBD. The three mAbs also competed with each other to bind to the RBD of MERS-CoV, and mutant analysis showed that the three mAbs possessed overlapping but distinct epitopes. Of the three mAbs, m336 neutralized both pseudovirus and live MERS-CoV infectivity in cells with exceptional potency (m336 inhibited 90% MERS-CoV pseudovirus infection at a concentration of 0.039 g/mL, and neutralized live MERS-CoV with IC_95_ of 1 g/mL and IC_50_ of 0.07 g/mL). Residues in the RBD crucial for m336 binding were L506, D510, E536, D539, W553, and V555 [14]. In vivo study demonstrated that prophylaxis with m336 reduced virus titers in the lung of rabbits infected with MERS-CoV [15], and m336 also provided transgenic mice expressing human DPP4 with full prophylactic and therapeutic protection from MERS-CoV [16]. However, another study with a non-human primate, the common marmoset showed that m336 could only alleviate the severity of the disease, and did not provide complete protection against MERS-CoV [17].

### 4.3. LCA60

IgG+ memory B cells were isolated from a MERS patient, and were subsequently immortalized with Epstein–Barr virus. A neutralizing mAbs, LCA60, was identified, and was the first fully human neutralizing mAb with naïve heavy and light chain pairs [18]. LCA60 efficiently neutralize MERS-CoV infectivity in cells. In vivo study showed that LCA60 provided BALB/c mice transduced with adenoviral vectors expressing human DPP4 (hDPP4) with both prophylactic and postexposure protection against MERS-CoV. Furthermore, the neutralizing efficacy of LCA60 was evaluated in IFN-α/β receptor-knockout mice that were more stringent models of MERS-CoV infection. After transducing with hDPP4, these mice showed more profound clinical symptoms when challenged with MERS-CoV [19]; administration of LCA60 reduced MERS-CoV titer in the lungs of these mice more effectively (lung viral titer reduced by three logs in one day for IFN-α/β receptor-knockout mice vs. three days for BALB/c mice) [18]. With naïve heavy and light chain pairs, LCA60 was more potent than 3B11 and comparable to m336. Cross-competition experiment demonstrated that LCA60 competed with 3B11 to bind to the RBD. LCA60 interacted with RBD residues around K493, and the LCA60 footprint on the RBD was partially overlapped with that of DPP4. Four residues in the RBD affected the binding of LCA60—namely T489, K493, E536, and E565—which were conserved in all MERS-CoV isolates. Moreover, compared with DPP4, the binding affinity of LCA60 to RBD was significantly higher (~500-fold). Therefore, one major neutralization mechanism of LCA60 was to competitively inhibit the interaction of the RBD with DPP4. Interestingly, virus escape studies demonstrated that under the selective pressure of LCA60, a mutant variant (V33A) in the N-terminal domain (NTD) of MERS-CoV S1 subunit was also generated [18]. A GMP-approved cell line (LCA60.273.1) that expresses LCA60 in high concentrations has been established, highlighting its application as promising therapeutics against MERS-CoV infection [20].

### 4.4. REGN3051 and REGN3048

Hybridoma B cells producing neutralizing mAbs against the S protein of MERS-CoV were generated by immunizing humanized transgenic mice (VelocImmune mice) with DNA encoding the MERS-CoV S protein. Two fully human neutralizing mAbs, REGN3051 and REGN3048, were obtained [21]. The two mAbs bound with high affinity to distinct epitopes on the RBD of MERS-CoV, which were conserved during the natural evolution of MERS-CoV. Mutation as a result of selective pressure by one mAb should not affect the binding of the other mAb. REGN3051 neutralized a broad range of MERS-CoV isolates, the prototype EMC/2012 strain and all clinical mutants including A431P, S457G, S460F, A482V, L506F, D509G, and V534A. With the exception of V534A variant, REGN3048 achieved similar neutralizing activity. In vivo study demonstrated that REGN3051 and REGN3048 reduced MERS-CoV replication in humanized DPP4 mice in both prophylactic and therapeutic settings [21]. When evaluated in the common marmoset, both mAbs seemed to be more effective for prophylaxis rather than for treatment of MERS-CoV infection [22].

### 4.5. MCA1

An anti-MERS-CoV phage display antibody library was constructed with the peripheral B cells of a MERS survivor, and a human neutralizing mAb against MERS-CoV, MCA1, was identified [23]. MCA1 showed potent neutralizing activity against MERS-CoV in cell entry tests. In vivo, MCA1 completely inhibited the replication of MERS-CoV in common marmosets when administrated prophylactically or therapeutically. Structure analysis of the MCA1 Fab-RBD complex showed that MCA1 formed direct contacts with the receptor-binding site (RBS) subdomain on the RBD. Epitopes on the RBS critical for MCA1 binding were D510, W535, E536, D539, Y540, R542, and Q544. Superimposed structure analysis of MCA1-RBD and hDPP4-RBD complexes showed that the binding interface of MCA1 was largely overlapped with that of hDPP4. Therefore, the neutralizing mechanism of MCA1 was achieved by competing with DPP4 for binding to the RBD [23].

### 4.6. MERS-4 and MERS-27

Two potent human neutralizing mAbs, MERS-4 and MERS-27, were derived from a nonimmune human yeast display antibody library, which was constructed with spleen and lymph node polyadenylated RNA from normal humans [24]. MERS-4 and MERS-27 inhibited the entry of both pseudovirus and live MERS-CoV into cells. Mutant analysis suggested that MERS-4 and MERS-27 recognized distinct epitopes in the RBD of MERS-CoV, and the epitopes of MERS-27 might be located away from those that recognized by MERS-4. Mutant analysis also demonstrated that residues D455, L507, E513, R542, L545, S546, P547, G549, and S508 in the RBD were critical for MERS-4 binding, while only S508 was important for MERS-27 binding. Combined use of MERS-4 and MERS-27 demonstrated a synergistic effect against pseudotyped MERS-CoV. MERS-4 bound to the RBD with about 45-fold higher affinity than DPP4. The primary neutralizing mechanism of MERS-4 and MERS-27 was through blocking the binding of the RBD to DPP4 [24]. Further structural analysis showed that MERS-4 bound to unique epitopes and caused conformational changes in the RBD interface critical for accommodating DPP4, therefore indirectly disrupting the interaction between the two. Moreover, MERS-4 also demonstrated synergistic effects with m336 and 5F9 (a NTD-specific mAb). The special neutralizing mechanism made MERS-4 a valuable addition for the combined use of mAbs against MERS-CoV infection [25].

### 4.7. MERS-GD27 and MERS-GD33

Thirteen ultrapotent neutralizing mAbs, which all targeted the RBD of MERS-CoV were generated following a protocol for the rapid production of antigen-specific human mAbs [26]. Briefly, antibody-secreting B cells were isolated from the whole blood of a MERS patient, and the antibody genes were amplified and cloned into vectors to transfect human cell lines for mAb production. Of the 13 mAbs, MERS-GD27 and MERS-GD33 exhibited the strongest neutralizing activity against both pseudovirus and live MERS-CoV in cell infection tests. MERS-GD27 directly competed with DPP4 to bind to the RBD to DPP4, and the crystal structure of MERS-GD27 showed that its epitopes were almost completely overlapped with DPP4-binding sites. MERS-GD27 and MERS-GD33 recognized distinct epitopes on the RBD, and had a low level of competing activity. The combined use of the two mAbs demonstrated synergistic effects in neutralization against pseudotyped MERS-CoV. Mutant analysis demonstrated that residues L506, D509, V534, E536, and A556 on RBD were important for the neutralizing activity of MERS-GD27, and residue R511was critical for MERS-GD33 [26]. Moreover, In vivo study found that MERS-GD27 could provide both prophylactic and therapeutic protection for hDPP4-trangenic mice against MERS-CoV infection [27].

## 5. Camel Neutralizing mAbs

Dromedary camels exposed to MERS-CoV showed mild clinical signs but developed exceptionally potent neutralizing antibodies. Camelid species naturally produced heavy chain-only antibodies (HCAbs) [28], which are dimeric and devoid of light chains, and their antigen recognition region is solely formed by the variable heavy chains (VHHs) (also called nanobodies, Nbs). VHHs or Nbs have long complementarity-determining region 3 (CDR3) loops and are capable of binding to unique epitopes not accessible to conventional antibodies [29]. Notably, camelid VHHs are relatively stable and can be produced with high yields in prokaryotic systems [30]. Because of their small size; good tissue permeability; and cost-effective production, storage, and transportation [31,32,33], VHHs or Nbs have been gaining acceptance as antiviral agents.

### 5.1. Chimeric Camel/Human HCAb-83

A VHH complementary DNA library was constructed with the bone marrow of dromedary camels infected with MERS-CoV. Four VHHs (VHH-1, VHH-4, VHH-83, and VHH-101) with high neutralizing activity were identified by direct cloning and screening of the phage display antibody library [34]. The four VHHs competed for a single epitope that partially overlapped with the RBD-DPP4 interface. Mutant analysis showed that the four VHHs did not bind to the D539N variant, which was a critical residue on the RBD for DPP4 binding [8,35]. Therefore, these VHHs most likely neutralized MERS-CoV by blocking its binding to DPP4. Of the 4 VHHs, VHH-83 showed the best neutralizing activity and epitope recognition. VHH-83 efficiently blocked the entry of MERS-CoV into cells, and it also prophylactically protected K18 transgenic mouse expressing hDPP4 from MERS-CoV infection. To extend the half-life of VHH-83 in serum, it was linked to a human Fc domain lacking the CH1 exon to construct the chimeric camel/human HCAb-83, which showed similar neutralizing activity as VHH-83. The chimeric camel/human HCAb-83 was highly stable in mice and provided K18 mice with fully prophylactic protection against MERS-CoV infection [34].

### 5.2. NbMS10 and NbMS10-Fc

Alpacas were immunized with recombinant MERS-CoV RBD-containing a C-terminal human IgG1 Fc tag, and VHHs were amplified from their peripheral blood mononuclear cells to construct a VHH phage display library. A neutralizing Nb, NbMS10, which bound with high affinity to the RBD of MERS-CoV and blocked the binding of RBD to DPP4, was identified [36]. To extend its in vivo half-life, the human-Fc-fused version, NbMS10-Fc, was constructed. NbMS10 competed with DPP4 to bind to RBD, indicating that the binding site of NbMS10 on RBD overlapped with that of DPP4. The binding site of the NbMS10 on the RBD was mapped to be around residue D539, which is part of a highly conserved conformational epitope at the receptor-binding interface in almost all the natural MERS-CoV published to date. NbMS10 did not neutralize psuedotyped MERS-CoV bearing a mutation in D539, confirming that residue D539 was critical for NbMS10 binding. NbMS10 efficiently neutralized the cell entry of live MERS-CoV. Moreover, NbMS10 showed potent prophylactic and therapeutic efficacy in protecting hDPP4-transgenic mice against MERS-CoV infection [36].

## 6. Discussion

For their exceptionally high neutralization activity in vitro and in vivo, these newly identified neutralizing mAbs are promising candidate therapeutics against the infection of MERS-CoV. However, the use of a single neutralizing antibody bears the risk of selecting escape mutants, a fact that has been observed for LCA60 and other described antibodies [12,18,37]. Notably, the majority of these escape mutations had little impact on viral fitness and the interaction of DPP4 with the RBD [12]. Moreover, mutants of MERS-CoV during natural infection have also been reported [38]. Escape from neutralization is a major concern with therapeutic neutralizing mAbs, however, this potential problem can be solved by combining mAbs that target distinct epitopes and show different neutralizing mechanisms [37]. This strategy can take advantage of the synergistic effects while decreasing the possibility of viral escape.

Currently, most of the MERS-CoV neutralizing mAbs compete with DPP4 binding to the RBD, and residues on the RBD critical for mAb neutralization are identified by mutant analysis. Almost all of the residues identified critical for mAb neutralization are located in RBM, and overlap with those critical for DPP4 binding (Figure 1B). With the availability of crystal structure of mAb Fab-RBD complex, the neutralization mechanism of these mAbs will be better illustrated.

Based on the crystal structure of RBD-DPP4, it was found that several conserved residues in the RBD are critical for the interaction of the RBD with DPP4 (Y499, L506, D510, E513, E536, D537, D539, Y540, R542, W553, and V555) [35,39]. Development of therapeutic neutralizing mAbs targeting those critically conserved residues might be important for combating MERS-CoV. Moreover, a study found a mouse-derived neutralizing mAb, 5F9, which bound to a possible linear epitope in the NTD of the MERS-CoV S1 subunit, exhibited efficient neutralizing activity against pseudovirus and live MERS-CoV in cell entry tests. This study highlighted the important role of NTD during the infection process of MERS-CoV. NTD might have significant implications for the development of prophylactic and therapeutic mAbs against MERS-CoV infection [40]. Although the in vitro neutralizing potency of 5F9 was approximately 10-fold lower than that of the RBD-targeting neutralizing mAbs [40], it may provide an alternative for the immunotherapy against MERS-CoV, once the virus mutates and is no longer susceptible to RBD-specific mAbs.

So far, there is a lack of appropriate animal models to mimic the pathology of MERD-CoV in humans. Commonly-used laboratory animals—such as wild-type mouse, ferret, hamster, and guinea pig—are not susceptible to MERS-CoV infection due to differences in critical amino acids in the S-binding domain of their DPP4 [41,42,43]. New Zealand rabbits, hDPP4-transduced/transgenic mice, camelids and non-human primates (rhesus macaque and common marmoset) are susceptible to MERS-CoV infection, however, rabbits showed asymptomatic infection [44]; dromedary camels displayed different clinical manifestations to that of humans [45]; rhesus macaque only showed transient lower respiratory infection [46], while common marmoset developed progressive pneumonia [47]; hDPP4-trangenic mouse expressed hDPP4 extensively, and resulted in multiple organ damage [48]; hDPP4-transduced mouse only exhibited mild transient clinical diseases [19]. With robust animal models, the protective effects of these neutralizing mAbs will be better evaluated. Furthermore, ongoing efforts on developing therapeutic neutralizing mAbs against MERS-CoV should also consider the different target populations (dromedary camels and humans) and their protective efficacy.
